# Diagnostic work-up and phenotypic characteristics of a family with variable severity of distal arthrogryposis type 2B (Sheldon-Hall syndrome) and TNNT3 pathogenic variant

**DOI:** 10.3389/fgene.2022.955041

**Published:** 2023-03-09

**Authors:** Ivana Dabaj, Robert Y. Carlier, Klaus Dieterich, Isabelle Desguerre, Julien Faure, Norma B. Romero, Wenting Trang, Susana Quijano-Roy, Dominique P. Germain

**Affiliations:** ^1^ APHP Université Paris-Saclay, Neuromuscular Unit, Department of Pediatric Neurology and ICU, Raymond Poincaré University Hospital (UVSQ), Garches, France; ^2^ Department of Neonatal and Pediatric Intensive Care, Charles Nicolle University Hospital, INSERM 1245, Rouen University, Rouen, France; ^3^ Nord-Est Ile de France National Neuromuscular Center, French Network (FILNEMUS) and European Reference Network (Euro-NMD), Paris, France; ^4^ APHP Université Paris-Saclay, Medical Imaging Department, Raymond Poincaré Universiy Hospital (UVSQ), Garches, France; ^5^ University Grenoble Alpes, Inserm, U1216, CHU Grenoble Alpes, Medical Genetics, Grenoble Institute of Neurosciences, Grenoble, France; ^6^ Assistance Publique-Hôpitaux de Paris, Paediatric Neurology Department - CHU Necker-Enfants-Malades, Paris, France; ^7^ Sorbonne Universités, UPMC University, INSERM UMRS974, CNRS FRE3617, Center for Research in Myology, Institut de Myologie, APHP GHU Pitié-Salpêtrière, Paris, France; ^8^ AnDDI-RARE Paris Referral Center for Birth Defects, Division of Medical Genetics, APHP Paris Saclay University, Paris, France; ^9^ University of Versailles, Division of Medical Genetics, Montigny, France

**Keywords:** Sheldon-hall syndrome, distal arthrogryposis type 2B, DA2B, *TNNT3*, neurogenic pattern

## Abstract

**Background:** Sheldon–Hall syndrome (SHS) or distal arthrogryposis 2B (DA2B) is a rare clinically and genetically heterogeneous multiple congenital contracture syndrome characterized by contractures of the distal joints of the limbs and mild facial involvement, due to pathogenic variants in genes encoding the fast-twitch skeletal muscle contractile myofiber complex (TNNT3, TNNI2, TMP2, and MYH3 genes).

**Patients and methods:** A 16-year-old boy with a history of congenital distal arthrogryposis developed severe kyphoscoliosis and respiratory insufficiency. His mother and younger sister had phenotypes compatible with SHS but to a much lesser extent. Diagnostic work-up included physical examination and whole-body muscular MRI (WBMRI) in all three patients and electroneuromyography (ENMG) and paravertebral muscle biopsy in the proband. DNA sequencing was used to confirm the diagnosis.

**Results:** Physical examination suggested the diagnosis of SHS. No muscle signal abnormalities were found in WBMRI. Large motor unit potentials and reduced recruitment suggestive of neurogenic changes were observed on needle EMG in distal and paravertebral muscles in the proband. DNA sequencing revealed a pathogenic variant in TNNT3 (c.187C>T), which segregated as a dominant trait with the phenotype.

**Discussion:** This is the first report on neurogenic features in a patient with DA2B and a pathogenic variant in TNNT3 encoding the fast-twitch skeletal muscle contractile myofiber complex. A superimposed length-dependent motor nerve involvement was unexpected. Whether developmental disarrangements in number, distribution, or innervation of the motor unit in fetal life might lead to pseudo-neurogenic EMG features warrants further studies, as well as the role of genetic modifiers in SHS variability.

## Introduction

Distal arthrogryposis (DA) is a group of rare clinically and genetically heterogeneous disorders primarily characterized by congenital contractures of the limb joints (Hall, 1997; Toydemir and Bamshad, 2009). The diagnostic criterion of DA is the presence of more than two of the following signs: camptodactyly, overlapping fingers and toes, ulnar deviation, thumb adduction, vertical talus and/or talipes equinovarus, and absent flexion finger creases. Some extreme cases can affect proximal joints or the spine with congenital hip dislocation, stiff elbows, and scoliosis. In 1996, the classification historically proposed by Hall (Hall et al., 1982) was revised and now recognizes ten different clinical subtypes of DA according to the proportion of shared features (Bamshad et al., 1996).

In addition to its clinical and genetic heterogeneity, DA is characterized by emerging mechanisms including lack of relaxation and calcium hypersensibility. Among distal arthrogryposis, DA2 is clinically divided into DA2A, also known as Freeman–Sheldon syndrome (FSS; OMIM #193700) and DA2B, also known as Sheldon–Hall syndrome (SHS; OMIM #601680), which are monogenic disorders with autosomal dominant inheritance distinguished on the basis of their facial features, including downward-slanting palpebral fissures, prominent nasolabial folds, a small mouth, a high-arched palate, a small mandible, and a characteristic triangular whistling-face in FSS (Robinson et al., 2007; Racca et al., 2015; Germain et al., 2018).

Whole-body muscle MRI findings have been correlated to genotype results in early-onset neuromuscular disorders, in particular congenital myopathies and congenital muscular dystrophies (Mercuri et al., 2005; Wattjes et al., 2010; Quijano-Roy et al., 2012). Moreover, WBMRI has recently been shown to be of particular interest in TPM2-related myopathies (Jarraya et al., 2012) an in certain forms of distal arthrogryposis such as DA10 (Stevenson et al., 2006) or arthrogryposis due to ECEL1 mutations (Dieterich et al., 2013) but has not been used in DA2B yet.

In this report, we describe the clinical features, molecular testing, and neuromuscular investigations of a family with Sheldon–Hall syndrome in an attempt to investigate the origin and pathophysiology of this distal arthrogryposis.

## Patients and methods

### Clinical cases

**FIGURE 1:**
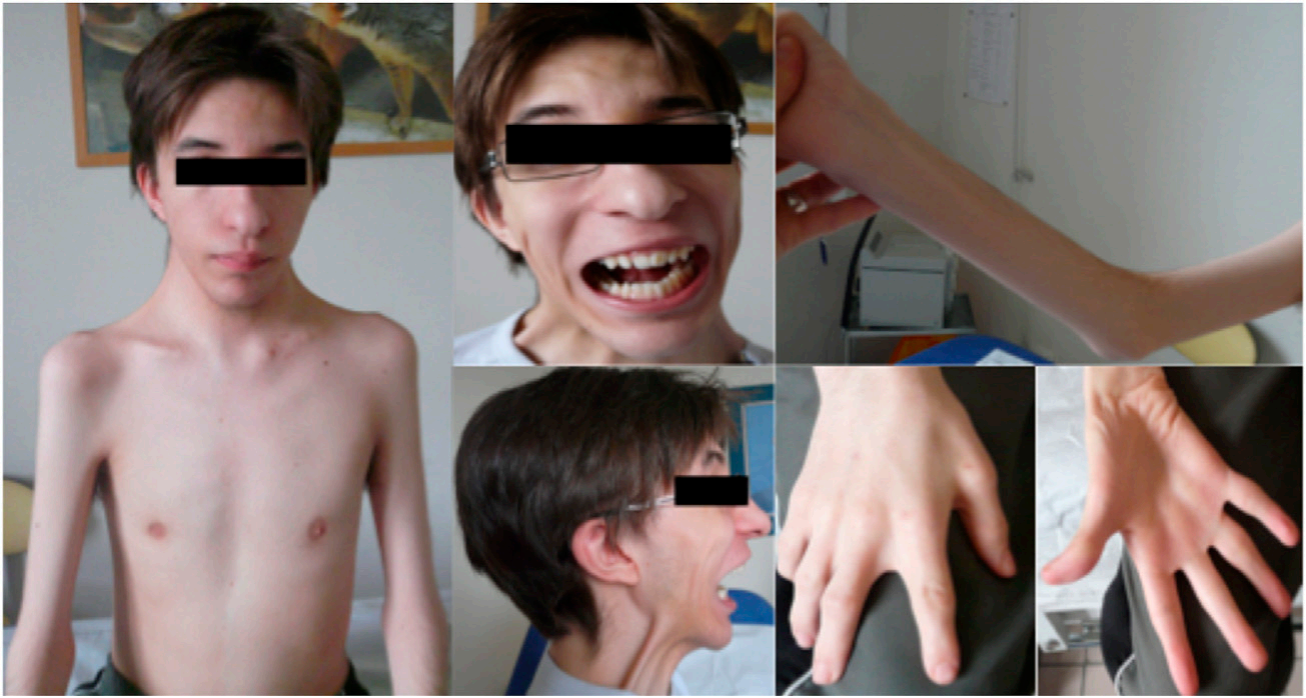
Clinical presentation of the index case and family tree.

We report on a French family with SHS over two generations in which affected individuals showed clinical variability. The index case presented with severe progressive kyphoscoliosis and a medical history of distal arthrogryposis. He was the oldest of all three children of non-consanguineous Caucasian parents and was born with clubfeet as well as hand and neck deformities (Figure 1)**.** He required orthopedic surgery during infancy for his hand and feet deformities. He developed scoliosis in early childhood (7 years) that was temporarily treated by bracing. He was subsequently lost to follow-up for 9 years before consulting in our hospital due to progression of his scoliosis. On examination at the age of 16, his height was 155 cm and weight 36 kg and had dysmorphic features including a triangular face, down-slanting palpebral fissures, mild epicanthus, a bulbous nose, marked nasolabial folds, and attached earlobes. Additional features included a small mouth with a high-arched palate, a mildly atrophic tongue with an irregular surface, a limited mouth opening, and prominent retrognathia (Figure 1). The patient had slight difficulties for upward ocular movements. The neck was short and showed pterygium coli. Mild webbing was also observed in the axillar region and between the fingers. The patient had mild elbow contractures. Camptodactyly and ulnar deviation of the fingers were present. The thumbs had limited abduction. Thenar and hypothenar atrophy was observed. There was distal limb atrophy and the patient left leg was hypotrophic and shorter compared to the right. The feet showed deformed with short and large toes, retracted Achilles tendon, and talipes equinovarus. Deep tendon reflexes were absent. There was neither tremor nor fasciculation on physical examination. The patient showed spinal stiffness and scoliosis. Respiratory function tests revealed a severe restrictive insufficiency with a forced vital capacity (FVC) of 1.200 L (30% of the normal theoretical values). A CT-scan revealed compression of left main bronchi due to the spinal lordosis at the thoracic level. Muscular strength was decreased in the proximal muscles and girdles, more marked in the shoulders (MRC score 4/5). Cardiac examination, including EKG and echocardiography, was unremarkable. Orthopedic treatment included thoracic pre-surgical casting and spinal fusion resulting in significant improvement in height (+11 cm) and pulmonary vital capacity (+600 mL).

The patient’s mother and younger sister had never previously sought medical advice. A clinical diagnosis of DA was made in our center on the basis of mild facial, hand, and spinal features. The proband’s mother showed dysmorphic features reminiscent of those observed in her son, particularly short stature, epicanthus, bulbous nose, prominent nasolabial folds, attached earlobes, and retrognathia but to a lesser extent. Mouth opening and vertical ocular movements were also limited. The neck was large and short but with no pterygium colli. She had spinal stiffness and mild kyphoscoliosis. She also exhibited camptodactyly, adducted thumbs, and thenar and hypothenar atrophy. Mild sternocleidomastoid hypotrophy with consequent mild weakness in neck flexion was noted.

The proband’s sister had a history of closed wrists at birth after pregnancy during which diminished fetal movements were reported. On physical examination, she had short stature (-3SD) but showed mild facial dysmorphic features (retrognathia, limited mouth opening, and short neck) together with spinal stiffness, mild kyphoscoliosis, and mild elbow contractures. She had hand deformities with marked adducted thumbs, finger retractions, and ulnar deviation. No major feet abnormalities were observed. Muscle strength was normal. The proband’s father and younger brother were not clinically affected.

### Methods

#### Electroneuromyography

ENMG evaluation was performed on the index patient before surgical intervention for scoliosis using a Keypoint portable machine (Dantec^®^, Natus Medical Incorporated, Middleton, WI, United States) and standard techniques (Gitiaux et al., 2015). NCS included motor median and tibial nerves and sensory median and sural nerves. Amplitudes, conduction velocity, and morphology of the compound motor action potentials (CMAPs) and sensory nerve action potentials (SNAPs) were analyzed.

Concentric needle ENMG was performed on two distal and one proximal muscles of the extremities (extensor digitorum brevis muscle in the lower limb; first dorsal interosseous and biceps brachialis muscles in the upper limb). ENMG activity at rest and under voluntary contraction was recorded for visual and automated analysis of the motor unit potential (MUP) morphology and recruitment. Repetitive nerve stimulation (3 Hz) was performed at rest on two muscles of the hand (abductor pollicis brevis and adductor digiti minimi) in order to exclude a neuromuscular junction disorder (Dieterich et al., 2013).

#### Whole-body magnetic resonance imaging

Images were acquired on a 1.5 T MRI system (Achieva release 11, Philips Medical Systems, Eindhoven, Netherlands) with a standard Q body coil, following the Garches protocol (Quijano-Roy et al., 2012). The index case was explored after surgery (titanium metallic implant compatible with the MRI machine). All three individuals were examined from head to toe in the supine position. The protocol included coronal and axial 6-mm-thick, non-enhanced T1-weighted Turbo Spin Echo images from head to toe divided into five areas (head/neck, chest/shoulder, abdomen/pelvis, thigh, and lower leg). The study was completed by the acquisition of STIR, T2-weighted sequences. None of the patients required sedation or ventilation. All T1 images were reviewed by a senior radiologist and an experienced neurologist to evaluate 109 muscles (54 pairs and the tongue) in each patient. The degree of fatty replacement of muscle fibers was assessed according to the distribution of abnormal signal intensity and was ranked from 1 to 4 as modified from Lamminen (1 normal, 2 mild, 3 severe, and 4 extreme or absent muscle) (Lamminen, 1990). A reduction in muscle volume was assessed independently of signal abnormality (Hankiewicz et al., 2015) and scored according to the relative muscle wasting in a particular region as follows: relatively preserved volume in comparison with muscles in the same region 0), mild reduction, 1) moderate reduction 2), severe reduction 3), and non-identifiable muscle 4). Muscle hypertrophy was observed in certain muscles but was not scored, since it was never associated with signal abnormalities and might be a secondary or compensatory phenomenon.

#### Spinal X-ray using the EOS imaging technique

Spinal X-ray was performed using the EOS 2D/3D X-ray imaging system to evaluate scoliosis while getting high-quality images with less radiation compared to standard imaging. The images were performed in a posteroanterior view.

#### Histopathological methods

Paravertebral muscle biopsy was performed on the proband during the surgical intervention for scoliosis. Histological analysis of the muscle was performed using staining with hematoxylin and eosin (H&E), Gomori trichrome (GT), and periodic acid–Schiff (PAS) reagent and histochemical reactions for nicotinamide adenosine dinucleotide-tetrazolium reductase (NADH-TR), myosin adenosine triphosphatase (ATPase) preincubated at pH 9.4, 4.6, and 4.3, and cytochrome c oxidase (COX) (BevilacquaBitoun et al., 2009).

## Results

### ENMG study

**FIGURE 2:**
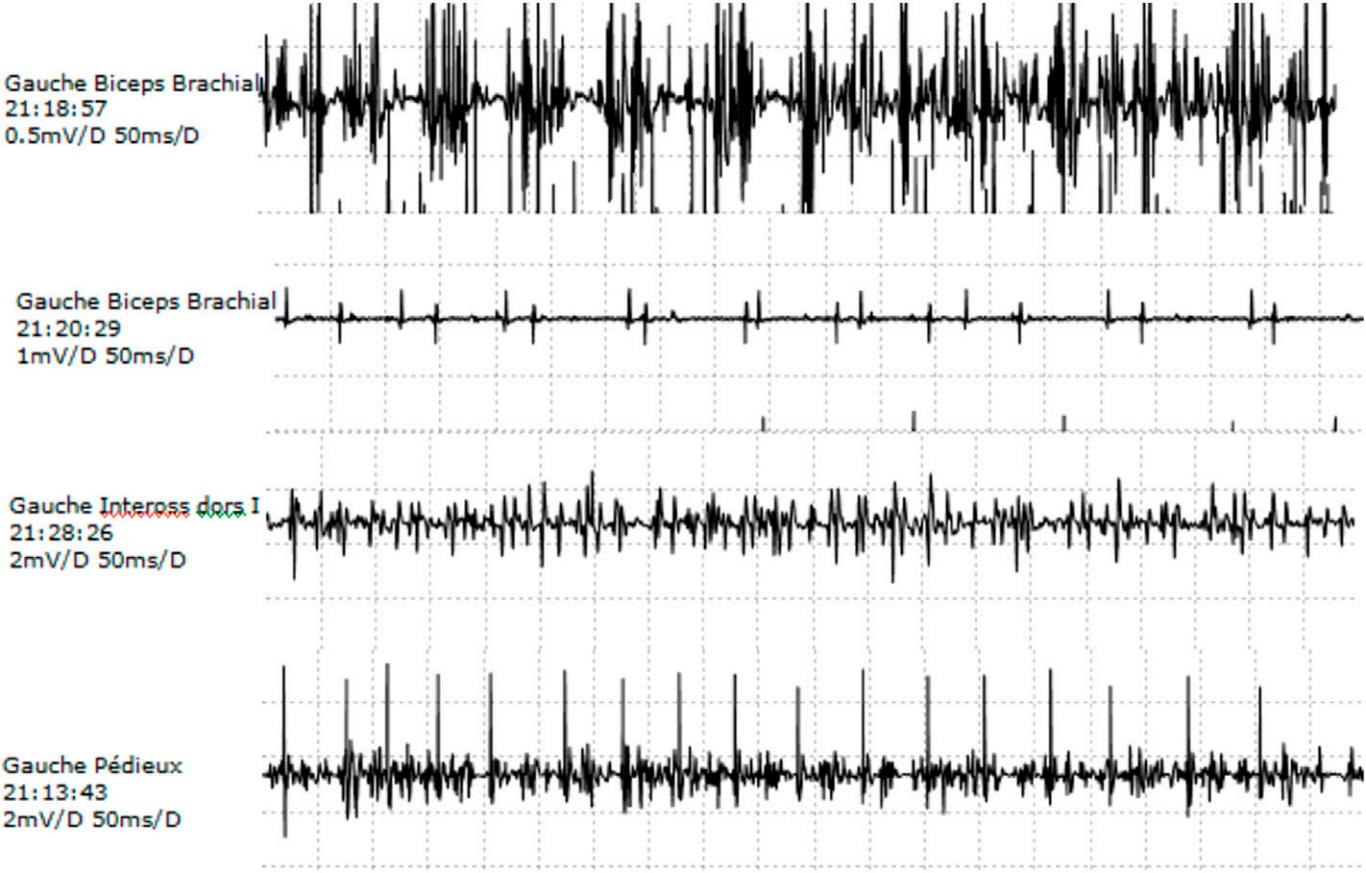
EMG. Needle EMG on left biceps, dorsal interosseus, and extensor digitorum brevis: the presence of large, polyphasic MUPs and decreased recruitment.

Motor and sensory NCS in lower and upper extremities were within normal values, although median CMAP was in the lower limit. Repetitive stimulation testing did not show any significant decrement at rest in the hand. Needle EMG was abnormal in all three muscles explored, with more prominent features in distal muscles of the upper extremity. No abnormal spontaneous activity was observed at rest. Voluntary contraction tracing showed large accelerated polyphasic MUPs, reaching 12.2 mv in APB, 6.8 mV in EDB, and 2.5 mV in biceps brachialis (normal values 1–1.5 mV). An increment in amplitude was more marked than the increase in duration. Reduced recruitment was also more prominent in distal muscles (Figure 2).

### Muscle imaging

**FIGURE 3:**
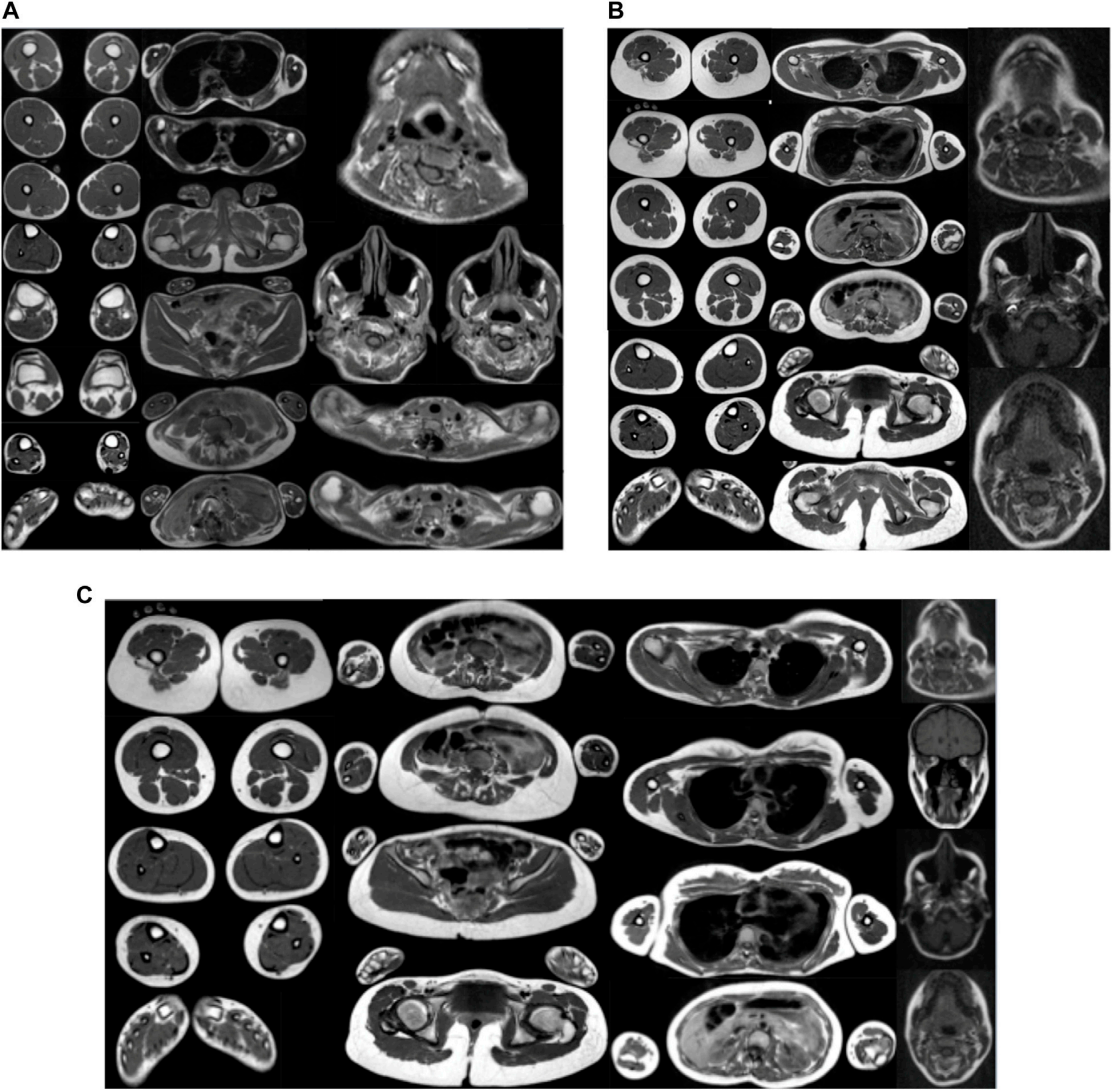
**(A)** WBMRI index case. Presence of muscular atrophy in lumbar and neck extensors, abdominal belt, and left calf muscles with no muscular fatty infiltration on the T1 sequence and no inflammatory changes on STIR. **(B)** WBMRI of the proband’s mother. Presence of bilateral muscular atrophy in lumbar, thoracic, and neck extensors, sternocleidomastoid, longus colli, and psoas and iliotibial tract with no muscular fatty infiltration on the T1 sequence and no inflammatory changes on STIR. **(C)** WBMRI of the proband’s sister. Presence of muscular atrophy in lumbar and thoracic extensors, sternocleidomastoid, longus colli, psoas and iliac, and temporal muscles with no muscular fatty infiltration on the T1 sequence and no inflammatory changes on STIR.

WBMRI performed on all three patients did not show any signal abnormality in the muscles examined (head, neck, trunk, shoulder and pelvic girdles, and upper and lower limbs), suggesting the absence of major fibro-adipose transformation of muscles in the disorder, even in the distal limb and temporomandibular joints. A signal abnormality cannot be excluded in the more severe index patient at the thoracic paravertebral level because of the presence of a metallic implant. As far as volume muscle abnormalities are concerned, the most marked atrophy was observed at the axial muscles, particularly at the lumbar and abdominal region. Other more selectively wasted muscles were the sternocleidomastoid, the temporal, and the ilio-psoas (Figure 3 and Table 1).

**TABLE 1:** Muscles showing atrophy on whole-body magnetic resonance imaging (WBMRI).

MRI abnormality	Index case	Mother	Sister
Atrophy (mild = +, moderate = ++, and severe = +++)	**Lumbar extensor (++): bilateral**	**Lumbar extensor (+): bilateral**	**Lumbar extensor (+): bilateral**
	**Thoracic extensor NA** ^a^	**Thoracic extensor (+): bilateral**	**Thoracic extensor (+): bilateral**
	Neck extensor (+): bilateral	Neck extensor (+): bilateral	Neck extensor: nl
	Sternocleidomastoid: nl	Sternocleidomastoid: L (++), R (+)	Sternocleidomastoid: R (+)
	Longus colli: nl	Longus colli (+): bilateral	Longus colli
	Abdominal belt (++): bilateral	Abdominal belt: nl	Abdominal belt: nl
	Psoas: nl	Psoas (+): bilateral	Psoas (+): bilateral
	Other	Other	Other: iliac R (++)
	Gastrocnemius lateral and medial head: L (+)	Iliotibial tract (+): bilateral	Temporal: L (+) and R (++)
	Soleus: L (+)		
	Tibialis anterior/posterior: L (+)		
	Extensor/flexor digitorum: L (+)		
	Peroneus muscles: L (+)		

^a^ Thoracic paravertebral muscles could not be evaluated due to previous surgical intervention for scoliosis (Left, L; Right, R; normal, nl).

### Spinal X-ray using the EOS imaging technique

**FIGURE 4:**
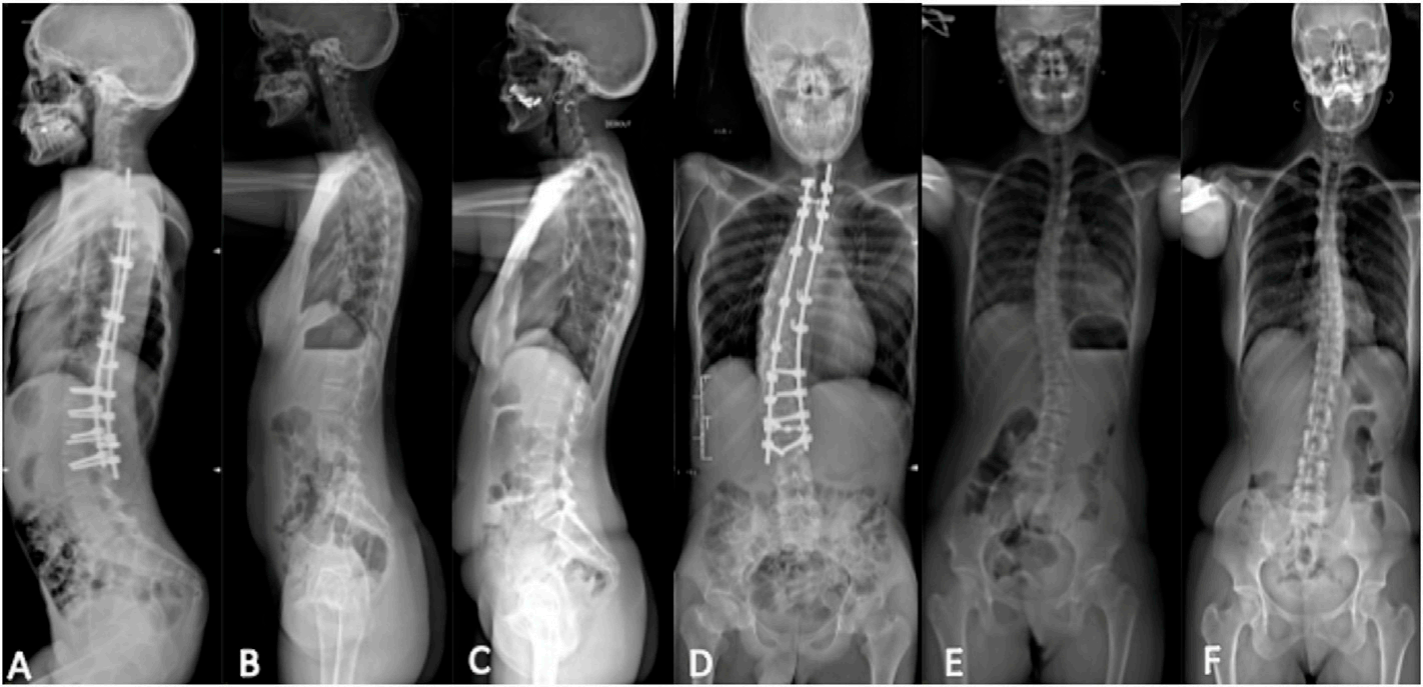
Spinal X-ray using the EOS technique. Lateral view: **(A)** index case, **(B)** sister, and **(C)** mother; posteroanterior view: **(D)** index case, **(E)** sister, and **(F)** mother. Thoracolumbar scoliosis with right convexity was observed from the atlanto-axial junction to L3 in the index case. X-ray showed thoracolumbar scoliosis with left convexity in the mother and sister.

Thoracolumbar scoliosis was detected in all three patients. In the index case, the deformity was severe and presented a right convexity. Orthopedic devices from the previous arthrodesis carried out all over the atlanto-axial junction to lumbar area (L3) prevented the visualization of the paraspinal areas. X-rays of the mother and sister showed a left convexity (Figure 4).

### Muscle biopsy

**FIGURE 5:**
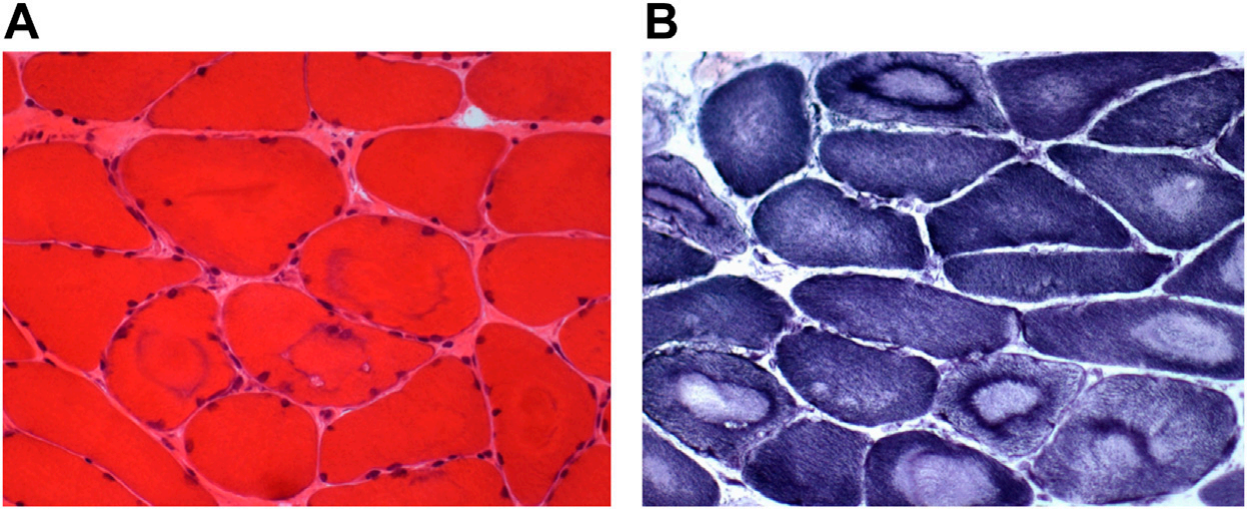
Muscle biopsy. Muscle sections from the index case showing a large variability of the fiber size [**(A)**, HE] and many « target fibers » with the oxidative reaction [**(B)**, NADH].

The biopsy from a paravertebral muscle showed fiber size variability. The large majority of muscle fibers appear as “target fibers” showing the typical concentric areas of disorganization corresponding to an ancient process of denervation/reinnervation. Using ATPase techniques, the distribution of fibers was not random. The sample was too small to clearly assess fiber type grouping distribution. Most fibers showed a fading of the ATPase staining in areas of “targetoid” appearance with a well-defined border (Figure 5).

### Molecular testing

DNA sequencing was carried out using the Sanger method to study all TNNT3 exons, intron–exon boundaries, and the promotor region. It revealed the presence of the c.187C>T; p. Arg63Cys missense variant in the *TNNT3* gene (NM_006757.4), previously reported as a mutational hotspot. The variant was confirmed to co-segregate with the DA2B phenotype in the three affected individuals and is considered pathogenic in the ClinVar database [NM_006757.4 (TNNT3):c.187C>T (p.Arg63Cys)].

## Discussion

Pathogenic variants in the genes encoding the fast-twitch skeletal muscle contractile myofiber complex, including troponin I2 (TNNI2), troponin T3 (TNNT3), tropomyosine 2 (TPM2), and embryonic myosin heavy chain 3 (MYH3), and the slow-twitch skeletal muscle myosin-binding protein C1 (MYBPC1) are known to cause either DA1, DA2A, or DA2B (Gurnett et al., 2009).

Since pathogenic variants that cause SHS are usually limited to a single amino acid or a single exon in throughout TNNT3, TNNI2, TMP2, and MYH3, we first screened these mutational hotspots as a reasonable diagnostic algorithm. Sequencing of proband’s DNA revealed a point mutation at nucleotide 187 (c.187C>T) in TNNT3 resulting in an arginine to cysteine substitution of the amino acid residue at codon 63 (p.Arg63Cys), which was also detected in the two other affected family members. A thorough review of the literature identified 45 patients in 12 unrelated kindreds, with a broad clinical spectrum ranging from severe fetal akinesia and congenital myopathy to distal arthrogryposis type 1 and distal arthrogryposis type 2B (Sung et al., 2003; Zhao et al., 2011; Beck et al., 2013; Daly et al., 2014; Sandaradura et al., 2018; De Burca et al., 2019; Reischer et al., 2020; Calame et al., 2021). The variant identified in our studied family (p.Arg63Cys) is a mutational hotspot recurrent mutation, and together with two other variants in the same amino acid residue (Arg63), they account for the vast majority of cases reported in the literature, Table 2 leading to DA2B and DA1 due to pathogenic variants in TNNT3 (c.187C>A: p. Arg63Ser (Beck et al., 2013); c.187C>T: p. Arg63Cys (Zhao et al., 2011; Beck et al., 2013); c.188G>A: p. Arg63His (Sung et al., 2003; Gurnett et al., 2009; Beck et al., 2013)). This amino acid residue is conserved in all known isoforms of TnT (Sung et al., 2003; Zhao et al., 2011; Beck et al., 2013; Daly et al., 2014; Sandaradura et al., 2018; De Burca et al., 2019; Reischer et al., 2020; Calame et al., 2021). This highlights the critical role of arginine at position 63 of the troponin T3 protein and suggests that a substitution has structural and/or functional consequences. In fact, alteration of the arginine at this position disturbs the interaction with tropomyosin, which in turn increases the number of calcium ions in the skeletal muscle and the contractility of the muscles and may result in the development of contractures and limb deformities (Robinson et al., 2007). The troponin complex, which consists of three regulatory proteins (troponin C, troponin I, and troponin T), contributes to muscle contraction in skeletal and cardiac muscle. Troponin T3 (TNNT3) is believed to be expressed only in skeletal muscle cells. Functional perturbation of the contractile apparatus of skeletal muscle during fetal development can cause multiple congenital contractures in individuals with an otherwise normal neuromuscular examination.

**TABLE 2:** Cases described in the literature with TNNT3 variants (Sung et al., 2003; Zhao et al., 2011; Beck et al., 2013; Daly et al., 2014; Sandaradura et al., 2018; De BurcaIoannou et al., 2019; Reischer et al., 2020; Calame et al., 2021).

Phenotype	Variant (cDNA)	Variant (protein)	Heterozygous/homozygous	Total number of cases	Reference
DA2B	c.188G>A	p. (Arg63His)	Heterozygous	5	Beck et al. (2013)
DA2B	c.188G>A	p. (Arg63His)	Heterozygous	3	Sung et al. (2003)
DA2B	c.188G>A	p. (Arg63His)	Heterozygous	18	Daly et al. (2014)
DA2B	c.188G>A	p. (Arg63His)	Heterozygous	4	De Burca et al. (2019)
DA2B	c.187C>T	p. (Arg63Cys)	Heterozygous	5	Zhao et al. (2011)
DA2B	c.187C>T	p. (Arg63Cys)	Heterozygous	2	Beck et al. (2013)
DA2B	c.525G>T	p. (Lys175Asn)	Heterozygous	1	Beck et al. (2013)
DA1	c.188G>A	p. (Arg63His)	Heterozygous	2	Beck et al. (2013)
DA1	c.187C>T	p. (Arg63Cys)	Heterozygous	2	Beck et al. (2013)
Congenital myopathy and DA	c.681 + 1G>A	N/A	Homozygous	1	Sandaradura et al. (2018)
Congenital myopathy and DA	c.681 + 1G>A	N/A	Homozygous	1	Calame et al. (2021)
Severe fetal akinesia syndrome	c.188G>A	p. (Arg63His)	Heterozygous	1	Reischer et al. (2020)

Since TNNT3 encodes the fast-twitch skeletal muscle contractile myofiber complex, the neurogenic pattern observed on EMG in the index patient, with abnormally large polyphasic motor unit potentials and reduced recruitment, was an unexpected finding. The absence of signs of acute denervation at rest and the distal predominance were in favor of a chronic motor neurogenic process. This, together with normal results of the sensory NCS, would suggest a chronic, length-dependent (distal), pure axonal motor neuropathy. The absence of signs of very active denervation makes unlikely (but does not rule out) a motor neuron disturbance. Neurogenic features have been described previously in several arthrogrypotic syndromes, particularly due to variants in TRPV4, BICD2, and DYNCH1H1 genes (Rossor and Reilly, 2021). Another possible explanation would be a “pseudo-neuropathic” pattern, which might be related to the developmental character of arthrogryposis itself. Peripheral nervous system causes of early immobility during development may be an abnormal development of not only joints and limbs of affected regions but also the motor unit elements. A compensatory phenomenon is observed in the preserved regions, compensatory enhanced collateral sprouting of the existing motor units, and a larger number of muscle fibers innervated cannot be excluded and should be further studied. However, the size of the MUPs observed was very much increased (2–8 times normal values), making it difficult to consider this as the only cause of the abnormalities observed. In addition, a muscle sample obtained from the paravertebral muscle of the index case during spine surgery revealed signs of denervation and reinnervation. These results must be considered carefully because paravertebral muscle findings are often difficult to interpret even in normal subjects.

WBMRI did not show significant fibro-adipose replacement in any muscles, including those clinically affected in distal limbs, although paravertebral muscles could not be explored in the proband, due to an artifact of the metallic implant. In the last decade, muscle MRI has appeared as a sensitive tool to investigate and map muscle involvement and to guide molecular diagnosis in patients with early-onset neuromuscular disorders, particularly in congenital myopathies and muscular dystrophies (Mercuri et al., 2005; Wattjes et al., 2010; Quijano-Roy et al., 2012). Recently, it has also been shown of particular interest in TPM2-related myopathies (Jarraya et al., 2012) and in certain forms of distal arthrogryposis such as that due to ECEL1 mutations (Dieterich et al., 2013). There is no previous report of muscle MRI studies and muscle pathology of patients affected with SHS. Interestingly, no signal abnormalities were identified in any of the three patients in the whole-body scanning, meaning absent or undetectable fibro-adipose replacement in muscles, but there was marked atrophy predominant in lumbar paravertebral muscles in all three patients. SHS may be considered in the differential diagnosis of myopathies associated with spinal rigidity and deformity, notably in patients with clinically mild distal involvement. In this context, WBMRI may be of great interest to distinguish SHS from myopathies associated with spinal stiffness (Mercuri et al., 2010; Quijano-Roy et al., 2012). In particular, in SEPN1-related myopathy, marked atrophy has been recently reported in axial muscles but was also present in other muscles of the body in a selective and recognizable profile associating muscle signal abnormalities, a consequence of fibro-adipose infiltration and selective muscle atrophy (Mercuri et al., 2010; Hankiewicz et al., 2015).

Progressive scoliosis and spinal deformity found in our patient could be the consequence of some denervation having started early during development, but it should be emphasized that these findings are not unusual in other forms of distal arthrogryposis and therefore deserve further attention and research. Studies on animal models (Toydemir and Bamshad, 2009) and the analysis and discovery of possible new genes inducing SHS phenotypes may also help to better understand how muscle or nerve development are affected in this heterogeneous disorder (Ju et al., 2013). In our patient, the hypotheses of an inherited disorder of connective tissue (Debette and Germain, 2014) were also ruled out.

Finally, the severity of clinical features was strikingly different between the index subject and his mother and sister. In line with our findings, intrafamilial variability was recently reported in distal arthrogryposis type 2B (De Burca et al., 2019). One limitation to our study is that whole-genome sequencing (WGS), which might have helped in clarifying for the presence of genetic modifiers responsible for intrafamilial variability, was not performed (Kimber et al., 2012).

## Conclusion

We described the neuromuscular and genetic investigations performed on a French Caucasian family with Sheldon–Hall syndrome (DA2B) and marked phenotypic intrafamilial variability. Sanger DNA sequencing revealed a missense pathogenic variant at one of the known mutational hotspots (c.187C>T; p. Arg63Cys) in the *TNNT3* gene. No major signal abnormalities in WBMRI were evidenced in any of the three individuals. The neurogenic changes were observed both on needle EMG and muscle biopsy of the index patient, warrantying further research on the precise pathophysiological mechanisms underlying Sheldon–Hall syndrome.

## Data Availability

The raw data supporting the conclusion of this article will be made available by the authors, without undue reservation.

## Abbreviations

DA, distal arthrogryposis; EMG, electromyography; EOS: FSS: Freeman–Sheldon syndrome; SHS, Sheldon–Hall syndrome or DA2B; TNN, troponin; TPM2, tropomyosine 2; MYH3, embryonic myosin heavy chain 3; MYBPC1, myosin-binding protein C1.

## References

[B1] Bamshad M. Jorde L. B. Carey J. C. (1996). A revised and extended classification of the distal arthrogryposes. Am. J. Med. Genet. 65 (4), 277–281. 10.1002/(SICI)1096-8628(19961111)65:4<277:AID-AJMG6>3.0.CO;2-M 8923935

[B2] Beck A. McMillin M. J. Gildersleeve H. I. S. Kezele P. R. Shively K. M. Carey J. C. (2013). Spectrum of mutations that cause distal arthrogryposis types 1 and 2B. Am. J. Med. Genet. A 161a (3), 550–555. 10.1002/ajmg.a.35809 23401156 PMC3581718

[B3] Bevilacqua J. A. Bitoun M. Biancalana V. Oldfors A. Stoltenburg G. Romero N. B. (2009). "Necklace" fibers, a new histological marker of late-onset MTM1-related centronuclear myopathy. Y. Acta Neuropathol. 117 (3), 283–291. 10.1007/s00401-008-0472-1 19084976

[B4] Calame D. G. Fatih J. Herman I. Akdemir Z. C. Du H. Jhangiani S. N. (2021). Biallelic pathogenic variants in TNNT3 associated with congenital myopathy. Neurol. Genet. 7 (3), e589. 10.1212/NXG.0000000000000589 33977145 PMC8105884

[B5] Daly S. B. Shah H. O'Sullivan J. Anderson B. Bhaskar S. Girisha K. M. (2014). Exome sequencing identifies a dominant TNNT3 mutation in a large family with distal arthrogryposis. Mol. Syndromol. 5 (5), 218–228. 10.1159/000365057 25337069 PMC4188168

[B6] De Burca A. Ioannou C. Vandersteen A. Pope F. M. Cilliers D. D. (2019). Intrafamilial variability of clinical features in distal arthrogryposis type 2B. Clin. Dysmorphol. 28 (1), 35–37. 10.1097/MCD.0000000000000243 30216196

[B7] Debette S. Germain D. P. (2014). Neurologic manifestations of inherited disorders of connective tissue. Handb. Clin. Neurol. 119, 565–576. 10.1016/B978-0-7020-4086-3.00037-0 24365320

[B8] Dieterich K. Quijano-Roy S. Monnier N. Zhou J. Fauré J. Avila D. (2013). The neuronal endopeptidase ECEL1 is associated with a distinct form of recessive distal arthrogryposis. Hum. Mol. Genet. 22 (8), 1483–1492. 10.1093/hmg/dds514 23236030

[B9] Germain D. P. Jurca-Simina I. E. (2018). “Principles of human genetics and mendelian inheritance,” in Neurometabolic hereditary Diseases of adults. Editor Burlina A. P. (Cham: Springer International Publishing), 1–28.

[B10] Gitiaux C. Blin-Rochemaure N. Hully M. Echaniz Laguna A. Calmels N. Bahi-BuissonLaugel V. (2015). Progressive demyelinating neuropathy correlates with clinical severity in Cockayne syndrome. Clin. Neurophysiol. 126 (7), 1435–1439. 10.1016/j.clinph.2014.10.014 25453614

[B11] Gurnett C. A. Alaee F. Desruisseau D. Boehm S. Dobbs M. B. (2009). Skeletal muscle contractile gene (TNNT3, MYH3, TPM2) mutations not found in vertical talus or clubfoot. Clin. Orthop. Relat. Res. 467 (5), 1195–1200. 10.1007/s11999-008-0694-5 19142688 PMC2664426

[B12] Hall J. G. (1997). Arthrogryposis multiplex congenita: Etiology, genetics, classification, diagnostic approach, and general aspects. J. Pediatr. Orthop. B 6 (3), 159–166. 10.1097/01202412-199707000-00002 9260643

[B13] Hall J. G. Reed S. D. Greene G. (1982). The distal arthrogryposes: Delineation of new entities-review and nosologic discussion. Am. J. Med. Genet. 11 (2), 185–239. 10.1002/ajmg.1320110208 7039311

[B14] Hankiewicz K. Carlier R. Y. Lazaro L. Linzoain J. Barnerias C. Quijano-Roy S. (2015). Whole-body muscle magnetic resonance imaging in SEPN1-related myopathy shows a homogeneous and recognizable pattern. Muscle Nerve 52 (5), 728–735. 10.1002/mus.24634 25808192

[B15] Jarraya M. Quijano-Roy S. Monnier N. Béhin A. Avila-Smirnov D. Carlier R. (2012). Whole-Body muscle MRI in a series of patients with congenital myopathy related to TPM2 gene mutations. Neuromuscul. Disord. 22 (2), S137–S147. 10.1016/j.nmd.2012.06.347 22980765

[B16] Ju Y. Li J. Xie C. Ritchlin C. T. Xing L. Hilton M. J. (2013). Troponin T3 expression in skeletal and smooth muscle is required for growth and postnatal survival: Characterization of Tnnt3 (tm2a(KOMP)wtsi) mice. Genesis 51 (9), 667–675. 10.1002/dvg.22407 23775847 PMC3787964

[B17] Kimber E. Tajsharghi H. Kroksmark A. K. Oldfors A. Tulinius M. (2012). Distal arthrogryposis: Clinical and genetic findings. Acta Paediatr. 101 (8), 877–887. 10.1111/j.1651-2227.2012.02708.x 22519952

[B18] Lamminen A. E. (1990). Magnetic resonance imaging of primary skeletal muscle diseases: Patterns of distribution and severity of involvement. Br. J. Radiol. 63 (756), 946–950. 10.1259/0007-1285-63-756-946 2268764

[B19] Mercuri E. Clements E. Offiah A. Pichiecchio A. Gessica Vasco G. Muntoni F. (2010). Muscle magnetic resonance imaging involvement in muscular dystrophies with rigidity of the spine. Ann. Neurol. 67 (2), 201–208. 10.1002/ana.21846 20225280

[B20] Mercuri E. Jungbluth H. Muntoni F. (2005). Muscle imaging in clinical practice: Diagnostic value of muscle magnetic resonance imaging in inherited neuromuscular disorders. S. Curr. Opin. Neurol. 18 (5), 526–537. 10.1097/01.wco.0000183947.01362.fe 16155435

[B21] Quijano-Roy S. Avila-Smirnow D. Carlier R. Y. WB- MRI muscle study group (2012). Whole body muscle MRI protocol: Pattern recognition in early onset NM disorders. Neuromuscul. Disord. 22 (2), S68–S84. 10.1016/j.nmd.2012.08.003 22980770

[B22] Racca A. W. Beck A. E. McMillin M. J. KorteBamshad S. F. M. J. Regnier M. (2015). The embryonic myosin R672C mutation that underlies Freeman-Sheldon syndrome impairs cross-bridge detachment and cycling in adult skeletal muscle. Hum. Mol. Genet. 24 (12), 3348–3358. 10.1093/hmg/ddv084 25740846 PMC4481580

[B23] Reischer T. Liebmann-Reindl S. Bettelheim D. Balendran-Braun S. Streubel B. (2020). Genetic diagnosis and clinical evaluation of severe fetal akinesia syndrome. Prenat. Diagn 40 (12), 1532–1539. 10.1002/pd.5809 32779773 PMC7756553

[B24] Robinson P. Lipscomb S. Preston L. C. Altin E. Watkins H. Ashley C. (2007). Mutations in fast skeletal troponin I, troponin T, and beta-tropomyosin that cause distal arthrogryposis all increase contractile function. Faseb J. 21 (3), 896–905. 10.1096/fj.06-6899com 17194691

[B25] Rossor A. M. Reilly M. M. (2021). Neurogenic arthrogryposis and the power of phenotyping. Neuromuscul. Disord. 31 (10), 1062–1069. 10.1016/j.nmd.2021.07.399 34736627

[B26] Sandaradura S. A. Bournazos A. Mallawaarachchi A. Cummings B. B. Waddell L. B. Jones K. J. (2018). Nemaline myopathy and distal arthrogryposis associated with an autosomal recessive TNNT3 splice variant. Hum. Mutat. 39 (3), 383–388. 10.1002/humu.23385 29266598 PMC5805634

[B27] Stevenson D. A. Swoboda K. J. Sanders R. K. Bamshad M. (2006). A new distal arthrogryposis syndrome characterized by plantar flexion contractures. Am. J. Med. Genet. A 140 (24), 2797–2801. 10.1002/ajmg.a.31528 17103435 PMC3244115

[B28] Sung S. S. Brassington A. M. E. Krakowiak P. A. Carey J. C. Jorde L. B. Bamshad M. (2003). Mutations in TNNT3 cause multiple congenital contractures: A second locus for distal arthrogryposis type 2B. Am. J. Hum. Genet. 73 (1), 212–214. 10.1086/376418 12865991 PMC1180583

[B29] Toydemir R. M. Bamshad M. J. (2009). Sheldon-Hall syndrome. Orphanet J. Rare Dis. 4, 11. 10.1186/1750-1172-4-11 19309503 PMC2663550

[B30] Wattjes M. P. Kley R. A. Fischer D. (2010). Neuromuscular imaging in inherited muscle diseases. Eur. Radiol. 20 (10), 2447–2460. 10.1007/s00330-010-1799-2 20422195 PMC2940021

[B31] Zhao N. Jiang M. Han W. Bian C. Li X. Huang F. (2011). A novel mutation in TNNT3 associated with Sheldon-Hall syndrome in a Chinese family with vertical talus. Eur. J. Med. Genet. 54 (3), 351–353. 10.1016/j.ejmg.2011.03.002 21402185

